# Abdominoplasty for treatment of abdominal gun-shot wound sequalae – A case report

**DOI:** 10.1016/j.ijscr.2020.06.056

**Published:** 2020-06-18

**Authors:** Vladislav Pavlovich Zhitny, Noama Iftekhar, Shannon Moreno, Frank Stile

**Affiliations:** a School of Medicine, University of Nevada, Las Vegas, Las Vegas, NV, USA; b School of Medicine, Loyola University of Chicago, Maywood, IL, USA; c Stile Aesthetics, Las Vegas, NV, USA

**Keywords:** Abdominoplasty, Tummy tuck, Scar revision, Gunshot wound

## Abstract

**Background:**

Gun violence is a public health epidemic in the United States with 15,315 non-suicide related deaths reported in 2019 by the Gun Violence Archive. To date, abdominoplasty is primarily a cosmetic procedure, which involves low-lying incision, removal of excess skin, fat, and tissue, and strengthening of the abdominal wall musculature. There currently are limited reports in regard to abdominoplasty as a procedure for scar revision and abdominal deformity repair associated with gunshot related injuries.

**Methods:**

An African American female patient, 38 years of age, presented for correction of a wide healed surgical incision and incisional hernia in the midline abdomen. This resulted from a trauma laparotomy in treatment of multiple gunshot wounds.

**Results:**

Patient presented post-operatively with no complications. She had successful reduction of her incisional hernia using an anatomic repair. Her wide hypertrophic abdominal scar was excised and primarily closed.

**Conclusion:**

In the cases of abdominal hernia and scarring secondary to trauma laparotomies performed for gunshot wounds, abdominoplasty is an option for revision and repair.

## Highlights

• Limited literature exists on abdominoplasty for therapeutic purposes.
• Patient arrived with emotional distress related to lasting gunshot injury scar.
• Abdominoplasty was completed for revision of the gunshot injury.
• Plastic surgeons should be creative in their techniques for revision.

## 1 Introduction

Abdominoplasty is a popular cosmetic surgery procedure with 130,081 tummy-tucks performed in 2018 in the United States [2]. This high demand procedure is often marketed as part of the “mommy-makeover” packages. While abdominoplasty is primarily a cosmetic surgery, it does offer therapeutic benefit from improved self-image. The surgery corrects a drooping abdomen, which is often the result of stretched skin, a localized accumulation of fat and a weakened abdominal wall.

This case report examines a female patient who suffered gunshot wounds to the abdomen one year prior. Gun violence is a public health epidemic in the United States with 15,315 non-suicide related deaths reported in 2019 by the Gun Violence Archive [1]. The trauma laparotomy that was performed to save her life, resulted in an obvious hypertrophic and hyper-pigmented scar in the midline abdomen as well as an incisional hernia in her epigastric region. An abdominoplasty procedure was employed to excise the scar, reduce the hernia and strengthen the weakened abdominal musculature. This case has been reported following the SCARE (Surgical Case REport) Guidelines [3].

## 2 Case-presentation

**Fig. 1:**
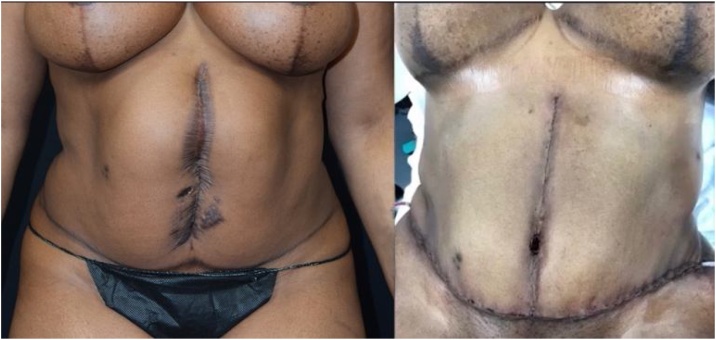
Ventral View a) Before: Large Surgical Abdominal Scar Status Post-Gunshot Wounds b) After: Abdominoplasty, Hernia Repair and Excision of Hypertrophic Scar Tissue.

A 38-year-old African American female presented to clinic for evaluation of a hypertrophic and hyper-pigmented scar and hernia in the ventral-midline abdominal area (Fig. 1). The patient had been a victim of gun-violence. She was shot several times in her torso approximately one year prior to this visit. During her emergency trauma procedure, a surgeon performed a laparotomy. She was found to have multiple small bowel injuries and a ruptured spleen. Small bowl repair and splenectomy were performed. The wound was left open and allowed to heal by secondary intention.

Significant past medical/surgical history included blood transfusion during pregnancy, liposuction, breast lift, and a previous cosmetic abdominoplasty. On physical examination the patient was found to have a hypertrophic and hyper-pigmented scar in the midline abdomen as well as an incisional hernia in her epigastric region. Her belly was ptotic and she experienced neuropraxia in the region of the midline scar. The abdominal musculature including rectus abdominis muscles appeared fully intact without any obvious atrophy or impaired function. There was an obvious diastasis.

Since the trauma, the patient experienced significant discomfort, distorted body image, anxiety and depression. Prior to the surgical procedure the patient, weighed 91.2 kg and her height was 1.73 m (Body mass index: 30.6 kg/m^2^). Patient’s pre-operative labs and echocardiography were normal. She was in over-all good health and was cleared for abdominoplasty surgery.

## 3 Operative procedure

**Fig. 2:**
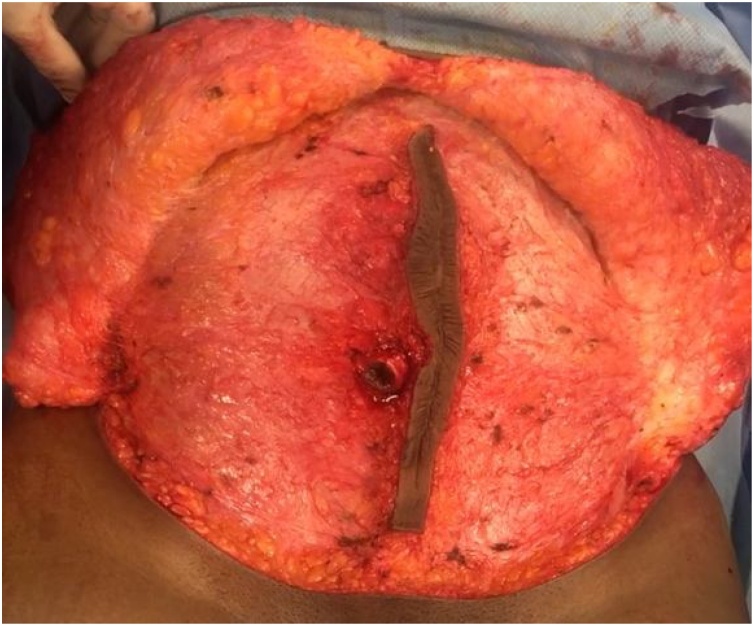
Dissection of the abdominal flap and the midline-scar tissue prior to excision with midline scar tissue island preserved.

The surgery was completed with the patient in the supine position using general anesthesia. A new abdominoplasty incision was created just distal to her original tummy-tuck incision. The midline scar was outlined, incised and de-epithelialized in its entirety. Next, dissection of the abdominal skin and fat flap from the abdominal wall fascia proceeded superiorly and laterally to the level of the umbilicus. The umbilicus was then carefully incised circumferentially and divided from the abdominal flap. Dissection of the abdominal flap then proceeded to the xiphoid process midline and costal margin bilaterally (Fig. 2). During this part of the dissection, a large epigastric incisional hernia was encountered. Great care was taken to skeletonize the hernia from the surrounding tissue without violating the hernia sac. The central island of de-epithelialized scar was carefully preserved.

**Fig. 3:**
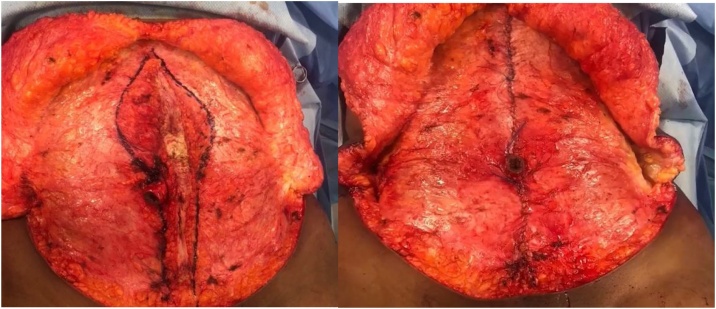
A) De-epithelialized scar tissue island. Note epigastric hernia in right upper aspect of diastasis outline. B)Re-approximation of the rectus abdominis fascia and reduction of the diastasis.

After outlining the lateral edges of the hernia defect, a series of 2−0 prolene sutures were used the reduce and repair the hernia. The rectus diastasis was next repaired beginning at the xyphoid using a double-looped nurolon stitch to reapproximate the fascia extending to the umbilicus. The lower part of the diastasis was reduced in a similar fashion from just below the umbilicus to the pubis (Fig. 3).

The free skin flaps were drawn inferiorly, and the redundant skin was excised. The distal edges were sutured to together at the midpoint of the abdominoplasty incision. The vertical scar and the abdominoplasty incision were closed using a series of interrupted 0 and 2–0 vicryl sutures. A new umbilicus position was marked and the umbilicus was brought out through the midline incision. It was secured using half-buried horizontal mattress nylon sutures.

A #7 flat Jackson-Pratt drain was placed on either side of the umbilicus and exited the lateral aspect of the incision bilaterally. After securing the drains a 4–0 monocryl suture was placed in a subcuticular fashion further and more precisely approximating the skin edges of both the horizontal and vertical incisions. The measured weight of the excised skin flaps was 698 g.

## 4 Results - follow-up

Patient was discharged home shortly after being recovered in the PACU of the ambulatory surgery center. The patient reported satisfactory elimination of scar symptoms. The drains were removed at post-operative days 7 and 14 respectively. She returned to normal activity a two weeks post op and full activity at 4 weeks including exercise and resistance training. Patient recovered well with no complications.

## 5 Discussion

Gun violence is an American public health epidemic. Compared to other high-income nations combined, the United States has a firearm homicide rate that is 24.9 times higher [4]. Of the 14,415 murdered due to guns in 2017, seventy-one were murdered from mass shootings [5]. Fifty-two women are murdered monthly by a partner with a gun [6,7]. These statistics are, indeed, alarming and do not cover the emotional and physical trauma experienced by survivors of these events. Victims of gun violence often suffer from the psychological impacts of their trauma for years to decades following. A cohort study of patient-reported outcomes found that in young adult survivors, 48.6% of those who found the event traumatic suffered from posttraumatic stress disorder (PTSD) [8]. Among those who had been discharged from the hospital with “minor” injuries, one-third screened positive for PTSD.

Abdominoplasty, known as the “tummy tuck,” is a highly efficacious and popular contouring procedure. Frequently requested by women following age-related, weight, or pregnancy related changes, abdominoplasty helps strengthen the abdominal muscles and remove excess fat and skin that contribute to a sagging abdomen. Abdominoplasty remains the fifth most common cosmetic surgical procedure with a 48% growth rate since 2000 [2]. A retrospective study reported a 94.4% satisfaction rate among obese patients, and another study reported that 88.8% of patients were satisfied with their procedure [9,10]. A prospective study completed over a 13-year period in Germany observed significant improvements in self-esteem, life-satisfaction, and independence [11].

Plastic surgery procedures are highly valued for purposes of elective cosmetic enhancement, the psychological benefits are evident. While this field has always had roots in trauma care, few cases exist pertaining to abdominoplasty for revision and repair of gun-shot wound scarring. A patient’s scar, particularly one that is midline and ventral, can not only affect self-esteem based on its cosmetic appearance but also acts as a reminder to the trauma as discussed previously. Thus, abdominoplasty can offer both therapeutic and cosmetic assistance to survivors of gunshot trauma.

## 6 Conclusion

With the rising numbers of gun violence related events, it is imperative for physicians to construct solutions that empower and support survivors. Despite limited literature on the subject, abdominoplasty can be used for scar revision purposes related to traumatic injuries to the abdomen.

## Declaration of Competing Interest

No disclosures.

## Sources of funding

There are no sponsors for this study.

## Ethical approval

Exempt as this is a case study and incorporates less than 2 patients.

Approval has been given – patient signed the waiver/media release form.

## Consent

This is a case study. All relevant characteristics that could be identifiable have been removed or changed. Written informed consent was obtained from the patient for publication of this case report and accompanying images. A copy of the written consent is available for review by the Editor-in-Chief of this journal on request.

## Author contribution

Vladislav Zhitny came up with the concept. Vladislav Zhitny and Noama Iftekhar evenly contributed to the literature search and wrote the article. Shannon Moreno conducted the final review of the document. Frank Stile is the operating surgeon and conducted final review of the document.

## Registration of research studies

N/A as this is not a study.

## Guarantor

Dr. Frank L Stile.

## Key learning points

Abdominoplasty corrective procedure can be used in the care of gunshot wound victims with abdominal trauma.

## Provenance and peer review

Not commissioned, externally peer-reviewed.
